# Asian Pigeonwing Plants (*Clitoria ternatea*) Synergized Mesenchymal Stem Cells by Modulating the Inflammatory Response in Rats with Cisplatin-Induced Acute Kidney Injury

**DOI:** 10.3390/ph15111396

**Published:** 2022-11-13

**Authors:** Fatmah A. Safhi, Salha M. ALshamrani, Areej S. Jalal, Nabil S. Awad, Hussein Sabit, Fathy Elsayed Abdelgawad, Sama S. Khalil, Dina M. Khodeer, Maysa A. Mobasher

**Affiliations:** 1 Department of Biology, College of Science, Princess Nourah bint Abdulrahman University, Riyadh 11671, Saudi Arabia; 2 Department of Biology, College of Science, University of Jeddah, Jeddah 21959, Saudi Arabia; 3 Department of Genetics, Faculty of Agriculture and Natural Resources, Aswan University, Aswan 81528, Egypt; 4 College of Biotechnology, Misr University for Science and Technology, Giza 12563, Egypt; 5 Medical Biochemistry Department, Faculty of Medicine, Al-Azhar University, Cairo 11651, Egypt; 6 Chemistry Department, Faculty of Science, Islamic University of Madinah, Madinah 42351, Saudi Arabia; 7 Medical Physiology Department, Faculty of Medicine, Zagazig University, Zagazig 44519, Egypt; 8 Department of Pharmacology & Toxicology, Faculty of Pharmacy, Suez Canal University, Ismailia 41522, Egypt; 9 Department of Pathology, Biochemistry Division, College of Medicine, Jouf University, Sakaka 72388, Saudi Arabia

**Keywords:** *Clitoria ternatea*, Asian pigeonwing, cisplatin, mesenchymal stem cell, AKI, kidney disease

## Abstract

Acute kidney injury is a heterogeneous set of disorders distinguished by a sudden decrease in the glomerular filtration rate, which is evidenced by an increase in the serum creatinine concentration or oliguria and categorized by stage and cause. It is an ever-growing health problem worldwide, with no reliable treatment. In the present study, we evaluated the role of *Clitoria ternatea* combined with mesenchymal stem cells in treating cisplatin-induced acute kidney injury in rats. Animals were challenged with cisplatin, followed by 400 mg/kg of Asian pigeonwing extract and/or mesenchymal stem cells (106 cells/150 g body weight). Kidney functions and enzymes were recorded, and histopathological sectioning was also performed. The expression profile of IL-1β, IL-6, and caspase-3 was assessed using the quantitative polymerase chain reaction. The obtained data indicated that mesenchymal stem cells combined with the botanical extract modulated the creatinine uric acid and urea levels. Cisplatin increased the level of malondialdehyde and decreased the levels of both superoxide dismutase and glutathione; however, the dual treatment was capable of restoring the normal levels. Furthermore, all treatments modulated the IL-6, IL-1β, and caspase-3 gene expression profiles. The obtained data shed some light on adjuvant therapy using *C. ternatea* and mesenchymal stem cells in treating acute kidney injury; however, further investigations are required to understand these agents’ synergistic mechanisms fully. The total RNA was extracted from the control, the positive control, and all of the therapeutically treated animals. The expression profiles of the IL-6, IL-1β, and caspase-3 genes were evaluated using the real-time polymerase chain reaction. Cisplatin treatment caused a significant upregulation in IL-6. All treatments could mitigate the IL-6-upregulating effect of cisplatin, with the mesenchymal stem cell treatment being the most effective. The same profile was observed in the IL-1β and caspase-3 genes, except that the dual treatment (mesenchymal stem cells and the botanical extract) was the most effective in ameliorating the adverse effect of cisplatin; it downregulated caspase-3 expression better than the positive control.

## 1. Introduction

Acute kidney injury (AKI) is an emerging global healthcare issue without effective therapy [1,2]. It is characterized by a sudden rise in the blood creatinine or urine output. AKI occurs in around 10% to 15% of hospitalized patients, but more than 50% of intensive care patients have been found to have the condition [2]. It is one of a range of conditions summarized as acute kidney diseases and disorders (AKD). Because AKI can be lethal, kidney replacement therapy is frequently required [3,4]. The management of a patient with AKI depends on the clinical situation and the availability of resources. However, the efficacy of several frequently used treatments is still controversial. The evidence is significantly increased over the past decade when interventions are being combined [5]. Egypt and Saudi Arabia have a major chronic renal disease problem. Over 20,000 individuals are on dialysis in the Kingdom of Saudi Arabia, while 9810 are awaiting kidney transplants [6]. It is projected that 294.3 people out of every million inhabitants in Saudi Arabia are receiving some kind of renal replacement treatment.

Drug-induced nephrotoxicity is a significant and growing cause of AKI; it is the cause of AKI in approximately 20% of hospitalized patients with the disorder [7]. Cisplatin is a chemotherapeutic medication often used to treat solid tumors, such as ovarian, head and neck, and testicular germ cell malignancies. Cisplatin therapy is well known to cause AKI. Understanding the core pathophysiology of cisplatin-induced AKI is critical for creating less nephrotoxic cancer treatments. Using rodent models, the pathophysiology of cisplatin-induced AKI has been addressed mechanistically [8].

Several botanical extracts have been used to treat AKI, including *Cordyceps cicadae* [9], hesperetin [10], and curcumin [11], as well as *Clitoria ternatea* L. [12]. *C. ternatea*, commonly known as butterfly pea, has traditionally been used in Ayurvedic medicine. Various phytochemicals such as kaempferol, quercetin, myricetin glycosides, and anthocyanins have been isolated from the flowers. The plant’s flower is a good option for functional food applications due to its vast array of pharmacotherapeutic qualities and its safety and efficacy [13]. Its extracts are antibacterial, antipyretic, anti-inflammatory, analgesic, diuretic, local anesthetic, antidiabetic, insecticidal, blood platelet aggregation inhibiting, and smooth muscle relaxing [14]. This plant has been reported to have anti-inflammatory and antioxidant effects, and it has prevented l-NAME-induced renal injury and dysfunction in rats [12].

After sustaining a range of renal traumas, the kidney can heal itself. Mesenchymal stem cells (MSCs) have been proven to repair tissue damage caused by kidney injuries and illnesses [15,16]. MSC-induced regeneration is predominantly mediated by the paracrine release of soluble substances and extracellular vesicles such as exosomes and microvesicles [17]. Hypoxic mesenchymal stem cells (HMSCs) have become an innovative cell-based therapy in AKI. HMSCs, or HMSC-conditioned medium (HMSC-CM), can mitigate renal injury via modulating the tubular autophagy [18,19]. It is important to weigh out the challenges of obtaining MSCs in therapeutic settings, as well as the number of cells available and ethical constraints, before employing them to treat patients. Several researchers have tried different strategies to boost MSCs’ effectiveness. Enhancing MSCs’ immunomodulatory potential is crucial for increasing their therapeutic efficacy [20]. Medicinal plant extracts and MSCs show promise in stem cell and regenerative medicine. Medicinal plants may boost MSC cell treatment for noninfectious and infectious disorders [21].

In Saudi Arabia, butterfly pea flower tea is a common caffeine-free herbal tea, or tisane, beverage made from a decoction or infusion of the flower petals or even the whole flower of *Clitoria ternatea* (blue tea flowers or Asian pigeonwings). However, treating cisplatin-induced AKI with a dual treatment composed of Asian pigeonwing extract and MSCs has infrequently been discussed in contrast to other topics. Therefore, this is an important topic to study. Here, we aim to explore the effect of *C. ternatea* crude extract or MSCs, or a combination of them, on the amelioration of cisplatin-induced AKI in rats.

## 2. Results

### 2.1. Phytochemical Analysis

In the present study, Asian pigeonwing extract was used to mitigate cisplatin-induced renal injury in rats. The chemical composition of *Asian pigeonwing* samples performed using HPLC chromatograph and the GC-TSQ mass spectrometer is stated in Figure S1. The phytochemical analysis of the ethanolic extract is shown in Table 1.

### 2.2. Kidney Functions

**Figure 1:**
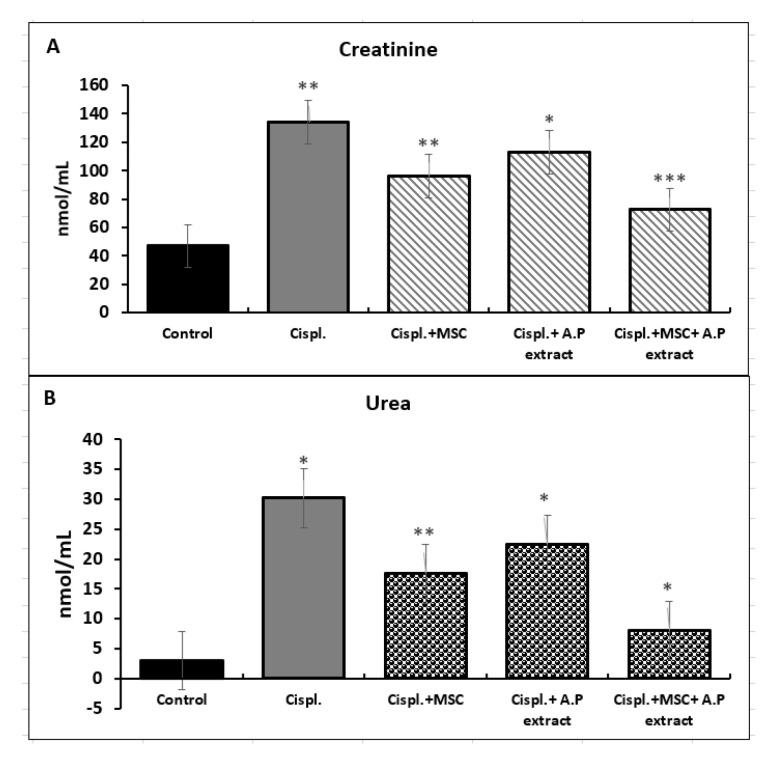

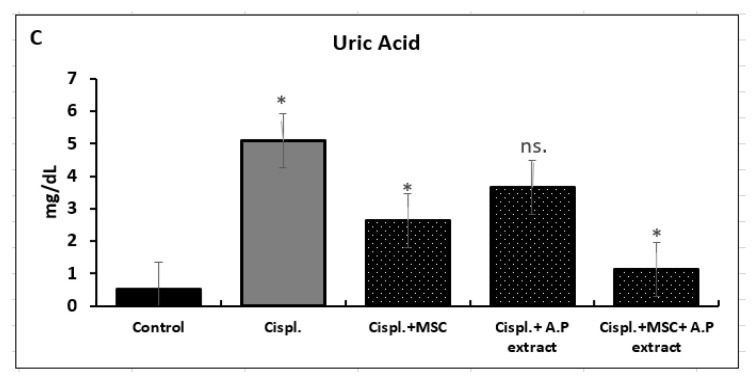
Kidney functions; creatinine, urea, and uric acid. C: control, Cispl.: positive control, Cispl. + MSC: cisplatin with mesenchymal stem cells, Cispl. + A.P extract: cisplatin with Asian pigeonwing extract, Cispl. + MSC + A.P: cisplatin with mesenchymal stem cells and Asian pigeonwing extract. (**A**) Creatinine, (**B**) urea, and (**C**) uric acid. Values are shown as mean ± SD and ns (*p* > 0.05), * (*p* < 0.05), ** (*p* < 0.01), *** (*p* < 0.001) compared to the corresponding value in the control group.

Albino rats were treated with cisplatin to induce kidney injuries for 10 days. During the induction period, animals were also treated with either Asian pigeonwing extract (400 mg/kg), MSCs (10^6^ cells/150 g BW), or both to alleviate the inflammatory effect of cisplatin. The kidney function was analyzed, emphasizing the creatinine, urea, and uric acid as indicators of the organ’s functionality. The data in Figure 1 indicate that the creatinine levels were significantly elevated when animals were challenged with cisplatin. Meanwhile, the treatments (BM-MSCs or Asian pigeonwing) after the induction of the kidney injuries revealed a significant decrease in the creatinine levels. The combined treatment of BM-MSCs plus Asian pigeonwing was the most effective treatment, followed by stem cells alone and the Asian pigeonwing extract. For the urea, the same profile was obtained. Although the stem cells alone or combined with the botanical extract significantly decreased the uric acid levels, the sole botanical extract did not show a significant decrease in its levels.

### 2.3. Oxidative Stress Markers

**Figure 2:**
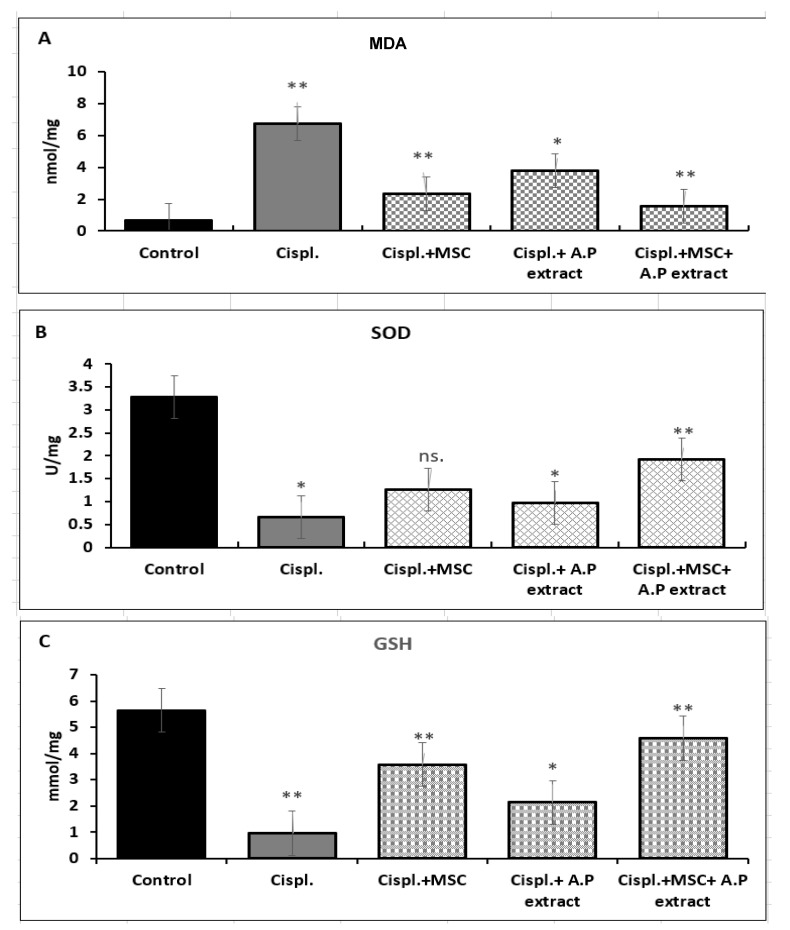
Oxidative stress markers MDA, SOD, and GSH. C: control, Cispl.: positive control, Cispl. + MSC: cisplatin with mesenchymal stem cells, Cispl. + A.P extract: cisplatin with Asian pigeonwing extract, Cispl. + MSC + A.P: cisplatin with mesenchymal stem cells and Asian pigeonwing extract. (**A**) MDA, (**B**) SOD, and (**C**) GSH. Values are shown as mean ± SD and ns (*p* > 0.05), * (*p* < 0.05), ** (*p* < 0.01) compared to the corresponding value in the control group.

To evaluate the oxidative stress upon cisplatin administration followed by different treatments, kidney malondialdehyde (MDA), superoxide dismutase (SOD), and glutathione (GSH) were measured in all animals. Rats challenged with cisplatin showed significantly elevated levels of MDA as compared with the control. Meanwhile, treating cisplatin-induced kidney injuries in rats with either the pigeonwing extract, stem cells only, or stem cells combined with the botanical extract showed a significant decrease in MDA levels. The MSCs combined with the botanical extract was the most effective treatment. SOD is a well-known enzyme that catalyzes the partitioning of superoxide radicals into molecular oxygen and hydrogen peroxide. The accumulation of superoxide causes severe damage to cells. Our data indicated that MSCs combined with the botanical extract caused a significant increase in the levels of SOD. The pigeonwing extract also showed a substantial increase in its levels, while the stem cells alone showed a nonsignificant increase. Glutathione can prevent damage caused by reactive oxygen species (ROS); therefore, it was a target of increase. The administration of cisplatin caused a severe reduction in the GSH levels in animals. In contrast, treatments caused a significant increase in their levels, with the dual hit being the most effective treatment (Figure 2).

### 2.4. Gene Expression Profiling

**Figure 3:**
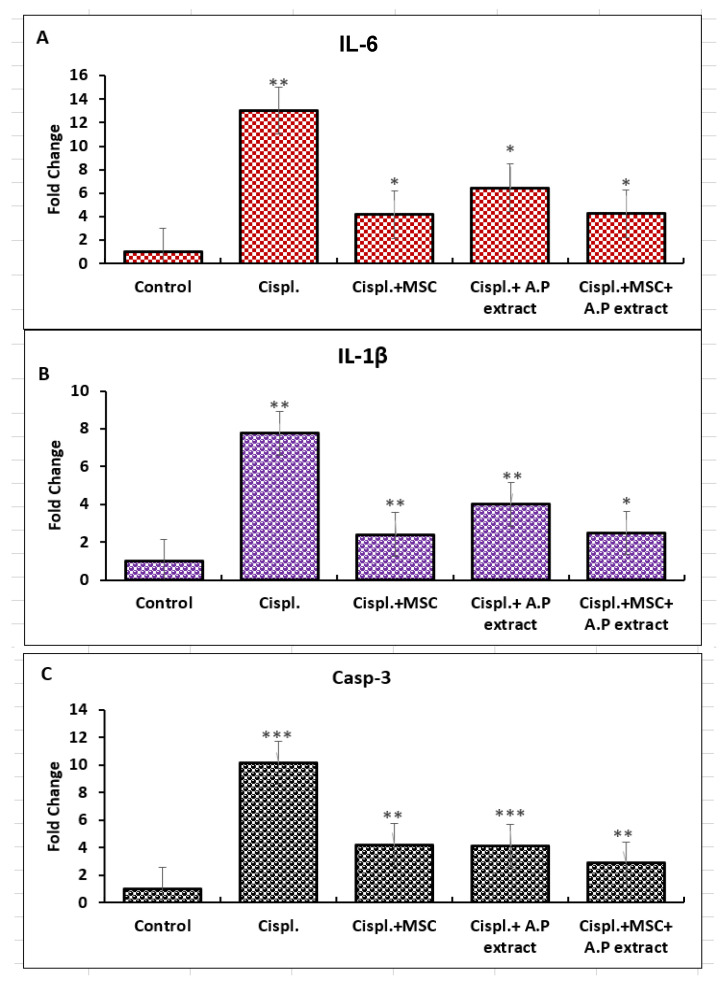
Gene expression profiles of IL-6, IL-1β, and caspase-3. C: control, Cispl.: positive control, Cispl. + MSC: cisplatin with mesenchymal stem cells, Cispl. + A.P extract: cisplatin with Asian pigeonwing extract, Cispl. + MSC. + A.P: cisplatin with mesenchymal stem cells and Asian pigeonwing extract. (**A**) IL-6, (**B**) IL-1β, and (**C**) caspase-3. Values are shown as mean ± SD (*p* > 0.05), * (*p* < 0.05), ** (*p* < 0.01), *** (*p* < 0.001) compared to the corresponding value in the control group.

The total RNA was extracted from the control, positive control, and all therapeutically treated animals. The expression profiles of the IL-6, IL-1b, and caspase-3 genes were evaluated using the real-time polymerase chain reaction (RT-PCR) (Figure 3). Cisplatin treatment caused a significant upregulation in IL-6 (12-fold). All treatments were able to mitigate the IL-6-upregulating effect of cisplatin, with the MSC treatment being the most effective. The same profile was observed in the IL-1b and caspase-3 genes, except that the dual treatment (MSCs and the botanical extract) was the most effective treatment in ameliorating the adverse effect of cisplatin, wherein it downregulated caspase-3 expression compared with the positive control.

### 2.5. Histopathological Sectioning

**Figure 4:**
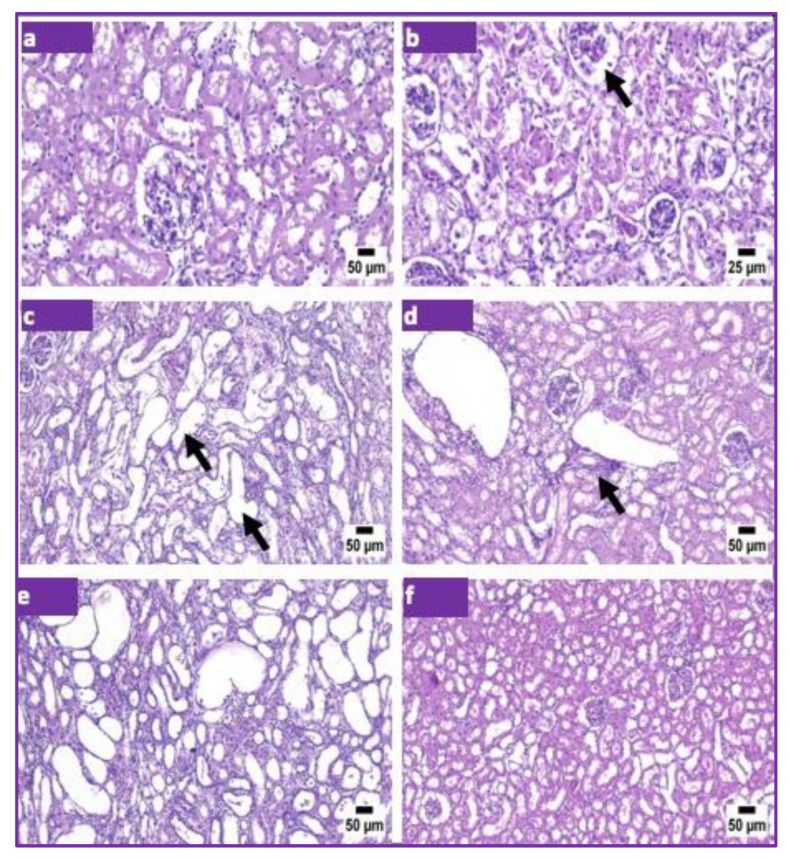
Histopathological analysis of kidneys of treated and untreated animals. (**a**) Photomicrograph of the kidney of the control group showing normal renal cortex. (**b**) Positive control group (received cisplatin) showing necrotic renal tubules. (**c**) Positive control group showing interstitial nephritis with cystically dilated tubules in the renal cortex (arrows). (**d**) The mesenchymal stem cell group showing interstitial nephritis. (**e**) The Asian pigeonwing group showing severe cystically dilated renal tubules associated with interstitial nephritis. (**f**) The Asian pigeonwing with mesenchymal stem cell group showing normal renal cortex.

In the present study, treated and untreated animals were anesthetized, sacrificed, and dissected according to the standards for laboratory animal care. The kidneys were removed, and histopathological sections were performed. Cells were stained with hematoxylin and eosin (H&E) staining (Figure 4). Figure 4a shows a normal kidney cortex. Figure 4b shows the positive control group (which received cisplatin) with necrotic renal tubules. Figure 4c shows the positive control group with interstitial nephritis with cystically dilated tubules in the renal cortex (arrows). Figure 4d shows animals treated with MSCs with interstitial nephritis. Figure 4e shows the Asian pigeonwing group with severely cystically dilated renal tubules associated with interstitial nephritis. Finally, Figure 4f depicts animals treated with the plant extract and stem cells with an apparently normal renal cortex.

## 3. Discussion

### 3.1. Renal Functions

Renal function was evaluated by assessing the levels of different markers, including creatinine, urea, and uric acid [22,23]. It is well established that cisplatin can cause acute kidney injury (AKI) and premature renal senescence [8,24,25]. The leading indicator of AKI is turbulence in the renal parameters, such as creatinine, uric acid, and urea. In the present study, these parameters were analyzed as indicators of chronic kidney disease (CKD) onset and progression. The data clearly showed a significant elevation of creatinine, uric acid, and urea in rats that were challenged with cisplatin. Animals were treated with the Asian pigeonwing extract, and this treatment caused a significant decrease in the levels of the three parameters assessed. Asian pigeonwing (*C. ternatea*) flower extract has been reported to have anti-inflammatory and antioxidant effects, as it was able to prevent L-NG-nitroarginine-methyl-ester-induced AKI in rats [26]. The proposed mechanism of action might be related to the downregulation of Nox4 expression and other oxidative stress events in rats [12]. Furthermore, MSCs can be seen as a novel approach to treating several diseases thanks to their high content of miRNAs [27,28].

Rat MSCs were administered to rats previously challenged with cisplatin to induce AKI in the present study. Our results indicated that stem cells modulated the renal function with significant values compared with the challenged animals. Studies have suggested that treating rats with urine-derived stem cells (USCs) decreased creatinine levels and renal tubular cell death, prevented inflammatory cell infiltration, and protected renal function. Additionally, exosomes isolated from USCs protected rats from ischemia/reperfusion-induced kidney injury [25]. Furthermore, MSCs alleviated AKI and reduced renal tubular injury, and when administered systemically, this treatment showed preferential homing to the proximal tubules in ischemic AKI rats [29]. A dual hit was tested to gain the most data on both treatments, and the results indicated its potent lowering effect on the parameters measured, which were creatinine, uric acid, and urea.

### 3.2. Renal Enzymes

As a reliable indicator of renal injury, we analyzed three renal enzymes, namely, SOD, GSH, and MDA, in treated and untreated animals. Cisplatin caused a significant decrease in the levels of SOD and GSH. At the same time, treatments with Asian pigeonwing extract or MSCs, or a combination of both treatments, restored the SOD and GSH levels somewhat toward normal. Although none of the treatments could restore the normal levels, the combined treatment was the most effective one, with significant differences compared with the cisplatin treatment.

It has been indicated that MSCs can alleviate AKI and damage in the renal interstitial capillary endothelial barrier via upregulation of AQP1 in the kidney [10].

Moreover, placental MSCs can alter the inflammatory environment by modulating the polarization of CD4+ T cells and macrophages, suppressing the pro-inflammatory factors IFN- and IL-17 and upregulating the expression of the anti-inflammatory factors TGF- and IL-10, ultimately resulting in kidney protection. Such functions may be mediated by the paracrine activity of placental-mesenchymal-stem-cell-derived extracellular vesicles (PMSC-EVs) [30].

Moreover, bone-marrow-derived MSCs can inhibit cell death in the kidney via upregulating SIRT1/parkin and activating mitophagy, ultimately mitigating AKI [31]. Other reports indicated that MSCs can protect against renal fibrosis caused by obstruction via downregulating STAT3 and upregulating STAT3-dependent MMP-9. These results demonstrated that they protected against obstruction-induced renal fibrosis, in part, by decreasing STAT3 activation and STAT3-dependent MMP-9 production [32].

It is believed that the paracrine effects of MSCs on renal healing, optimization of the microenvironment for cell survival, and inhibition of inflammatory responses result from their interaction with the injured kidney environment [33]. HMCSs may inhibit renal tubular apoptosis and mitigate renal impairment in rats with renal injury. This can provide additional mechanistic support for HMSC therapy and its evaluation in clinical studies of ischemic AKI [1].

Meanwhile, Asian pigeonwing extract also has a modulatory effect on cisplatin-induced renal injury. This extract was believed to prevent the production of fluorescent advanced glycation end products (AGEs) and protein oxidation in the bovine serum albumin/methylglyoxal (BSA/MG) system by decreasing the protein carbonyl concentration and avoiding protein thiol depletion. It also inhibited the oxidative breakage of DNA in the MG/lysine and 2,2′-azobis(2-methylpropionamidine) dihydrochloride (AAPH) systems by preventing superoxide anion and radical hydroxyl production. It is suggested that the fundamental processes responsible for preventing protein glycation and oxidative DNA damage are the direct carbonyl trapping ability and free radical scavenging activity of the extract [34]. Moreover, anthocyanins in Asian pigeonwings exhibit substantial in vitro and cellular antioxidant activity [35]. In the current study, we found that the protective effects of Asian pigeonwing extract related to the renin–angiotensin system (RAS). Asian pigeonwing extract inhibited RAS activation by directly decreasing ACE activity and increasing NO availability [36]. In vitro, the carboxylate and hydroxyl groups of phenolic compounds, the primary components in Asian pigeonwing extract, interact with the zinc ion at the ACE active site to suppress ACE function [37].

It is well known that damage to the structures of the kidneys may lead to a diminished level of renal function [38]. NO and Ang II regulate mesangial cell proliferation and death in glomerular microcirculation [39]. In addition, it has been shown that Ang II signaling is responsible for inducing renal ECM buildup through ROS activation [40]. This finding lends credence to the theory that oxidative stress plays a significant part in both the development of hypertension and the process that leads to kidney damage [41]. In addition, the putative molecular pathways implicated in the action of Asian pigeonwing extract on renal fibrosis might be mediated via the NF-αB pathway. Co-treatment with Asian pigeonwing extract seemed to inhibit the development of renal fibrosis and the buildup of glomerular extracellular matrix, according to the findings of the current investigation. According to a prior publication, this effect may have something to do with the ACE inhibitory action of the compound [42]. On the basis of our findings, administration of Asian pigeonwing extract to rats resulted in a decrease in Ang II levels. The extract from Asian pigeonwings lowered levels of an enzyme called Ang II, which in turn decreased production of another enzyme called Nox4 and oxidative stress.

## 4. Materials and Methods

### 4.1. Plant Sample Collection

Plant samples were collected and dried in the Aljouf region, Saudi Arabia. Samples of dried plants were ground into powder. Plant species were identified as *C. ternatea*, which belongs to the Fabaceae family, at the Faculty of Science of Aljouf University.

### 4.2. Preparation of Aqueous Extract

Five grams of powder from the plants’ leaves was extracted by 100 mL of distilled water at room temperature for 24 h. The distillate was centrifuged at 3000 rpm for 15 min and evaporated to near dryness, and the resulting viscous powder was dissolved in distilled water to obtain a stock solution [43].

### 4.3. Phytochemical Analysis and Assessment of Antioxidant Activity

The extract was phytochemically characterized by subjecting it to HPLC and gas chromatography–mass spectrometry (GC-MS) analysis. Then, we estimated the total phenolic compounds (TPCs) and total flavonoid compounds (TFCs) [44]. The TPCs, TFCs, and antioxidant power were estimated in the plant extracts. The TPCs were determined using the Folin–Ciocalteu reagent according to Singleton [45] and stated in milligrams per gram (mg/g) of gallic acid equivalent (GAE). The determination of flavonoids was carried out by the aluminum chloride method. The flavonoid contents were measured as the quercetin equivalent; it was used as the standard [46]. The ferric ion-reducing antioxidant power (FRAP) was determined to estimate the antioxidant power of each plant extract [47]; ascorbic acid was utilized as a positive reference standard.

### 4.4. Animals and Research Design

**Figure 5:**
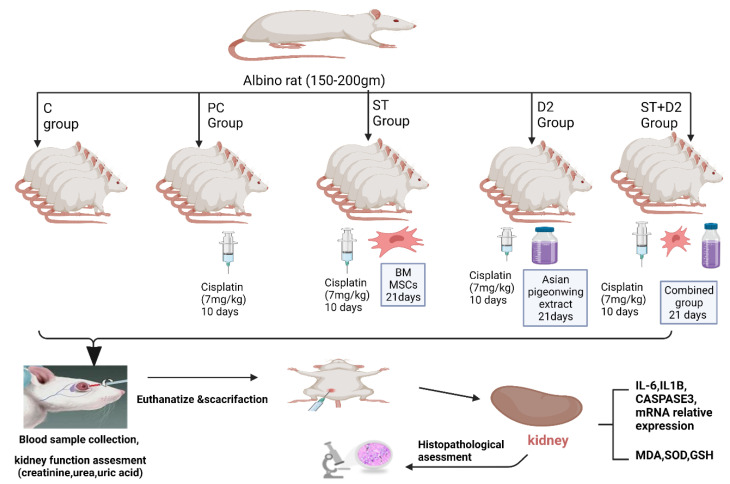
Summary of the animal model and the experimental design.

Twenty-five albino rats (150 to 200 g) were enrolled in the present study. Animals were grown under standard laboratory conditions with food and water offered ad libitum. Healthy males were randomly selected and divided into five groups (five rats each) as follows (Figure 5):

1. The control group received saline.
2. The cisplatin group was injected with a single dose of cisplatin of 7 mg/kg intraperitoneally (IP) to induce liver damage.
3. The BM-MSC group (bone-marrow-derived MSCs group) was injected with a single dose of cisplatin of 7 mg/kg IP, and on the next day began receiving 2 × 10^6^ BM-MSCs per day in phosphate buffer solution (PBS) by intravenous (IV) injection for 21 days.
4. The Asian pigeonwing group was injected with a single dose of cisplatin of 7 mg/kg IP, and on the next day began receiving 400 mg/kg of Asian pigeonwing extract (by oral gavage for 21 days).
5. The BM-MSC plus Asian pigeonwing group (combinational treatment group) was injected with a single dose of cisplatin of 7 mg/kg IP, and on the next day began receiving 2 × 10^6^ BM-MSCs in PBS by IV injection plus 400 mg/kg/day of Asian pigeonwing by oral lavage for 21 days.

Treatments were carried out for 21 consecutive days with BM-MSCs and/or Asian pigeonwing before the animals were sacrificed.

### 4.5. Stem Cells

Rat bone marrow MSCs were obtained from the Biochemistry and Molecular Biology Unit, Faculty of Medicine, Cairo University. The cell count per milliliter was adjusted to the rats’ body weight to reach the optimal dose of 10^6^ cells/150 g BW.

### 4.6. Blood and Tissue Sampling

Rats were euthanized by pentobarbital overdose (300 mg/kg body weight). Blood samples were collected, and the serum and kidney tissues were separated and used for further biochemical tests. Kidney tissue was quickly removed and divided into three sections. The first section was stored in Trizol reagent for real-time gene expression analysis at −80 °C. The second section was immersed in 15% formaldehyde solution for pathological examination. The third section was used to prepare a tissue homogenate and was stored at −80 °C. For the biochemical analyses, the tissue homogenate and blood serum were prepared immediately before starting the measurements.

### 4.7. Biochemical Analyses

Creatinine was measured using a creatinine (rat) ELISA Kit (Biovision, Catalog # E4370-100). Uric acid was estimated in blood serum using the uric acid enzymatic colorimetric method (Biodiagnostic kit, Cat. No. UA2120). MDA, SOD, and GSH were measured using tissue homogenate according to the manufacturer’s instructions in the biodiagnostic kit (Cat. No. MD2529, SD2521, and GR2511, respectively).

### 4.8. Histological Analysis

Formalin-fixed specimens were routinely dehydrated in ascending series of alcohol, cleared in xylol, and finally embedded in paraffin. Then, 4–5 µm thick tissues were sectioned and processed for H&E staining (Bancroft and Gamble 2008). Tissue slides were examined and were compared to their corresponding controls.

### 4.9. Gene Expression Analyses

Total RNA was extracted from treated and untreated animals, and one gram was converted to cDNA. The expression profiles of IL-1β, IL-6, and caspase-3 genes were analyzed. Primers used in the present study are presented in Table 2.

The expression of these genes was compiled using the StepOnePlus thermal cycler, and the thermal profile was 35 cycles composed of 45 s at 90 °C, 60 s at 58–60 °C, and 45 s at 72 °C. The cycles were followed by a 10 min of extension at 72 °C. The PCR products were determined by melting curve analysis for each primer pair to specify the amplification. β-Actin gene expression was measured as the internal housekeeping gene. The 2∆∆CT method was used to analyze the obtained data. Results were presented as fold change (RFC) relative to the negative control.

### 4.10. Statistical Analysis

All treatments were performed in triplicate, and data were analyzed by analysis of variance (one-way ANOVA test) using Tukey’s post hoc test; SPSS version 21 software was used for statistical analysis tests. Values were presented as mean ± SD.

## 5. Conclusions

AKI is a severe disease that has no reliable treatment. Several factors can initiate AKI. Herbal medicine could be one of the well-studied options to tackle such health issues, especially when it is combined with a naturally occurring biological agent such as the MSC. The treatment of AKI is ultimately the goal, wherein adjuvant therapy is the most promising approach. The present study indicated that using the combinatory therapy of Asian pigeonwing and MSCs restored the standard functions in cisplatin-treated rats. This approach suggests an important topic to study because of the promising results of the treatment. The limitation of this study is that the model is that cisplatin kidney injury may not be generalizable to other forms of acute kidney injury (e.g., secondary to sepsis or hypotension) and the number of samples have to be greater. However, further detailed studies are required for a more comprehensive understanding of the actual mechanism of action of the stem cells with such a botanical extract.

## Figures and Tables

**Table 1:** The phytochemical analysis of the Asian pigeonwing plant.

Total phenol (mg/g extract)	31.3
Flavonoid (mg/g extract, as quercetin)	5.2
FRAP assay reduction power (mg/g extract, as ascorbic acid)	25.8

**Table 2:** The primer sequences used in the present study.

Genes	Forward (5′–3′)	Reverse (5′–3′)	Size
IL-1β	CAGCAGCATCTCGACAAGAG	AAAGAAGGTGCTTGGGTCCT	123 bp
IL-6	AGTTGCCTTCTTGGGACTGA	CCTCCGACTTGTGAAGTGGT	126 bp
Caspase-3	GAGACAGACAGTGGAACTGACGA TG	GGCGCAAAGTGACTGGATGA	147 bp
β-Actin	GTGACATCCACACCCAGAGG	ACAGGATGTCAAAACTGCCC-	

## Data Availability

Not applicable.

## Supplementary Materials

The following supporting information can be downloaded at: https://www.mdpi.com/article/10.3390/ph15111396/s1, Figure S1: The chemical composition of *Asian pigeonwing*.
