# Supplementary material for: Asian Pigeonwing Plants (Clitoria ternatea) Synergized Mesenchymal Stem Cells by Modulating the Inflammatory Response in Rats with Cisplatin-Induced Acute Kidney Injury
Source: Pharmaceuticals (Basel). 2022 Nov 13;15(11):1396. doi: 10.3390/ph15111396 (PMC9695921; doi:10.3390/ph15111396)
Supplement: Supplementary file 1 [file pharmaceuticals-15-01396-s001.zip › pharmaceuticals-1958512-supplementary.pdf]

A)

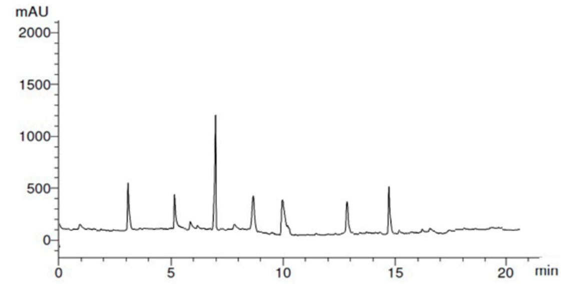

RT#	Compound	Concentration µg/ml
3.0	Chlorogenic	5.22
5.0	Syringenic	3.15
7.0	Cinnamic	11.65
8.7	Pyrogallol	3.64
10.0	Gallic	4.18
13.0	Ellagic	1.36
15.0	Benzoic	5.37

B)

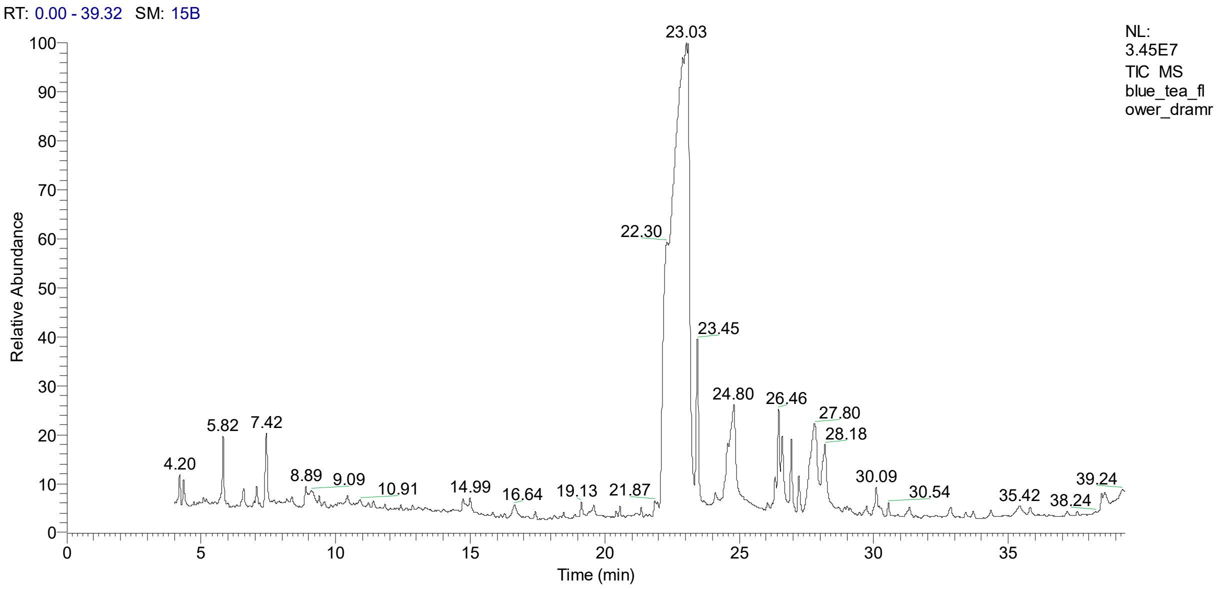

Compound number	Retention time	Compound name	Area%	Molecular weight	Molecular formula
1	4.21	Acetaldehyde, diisobutyl acetal	0.81	174	C10H22O2
2	4.34	4.34 BUTANE, 1,1'[METHYLENEBIS(OXY)]BIS-	0.85	160	C9H20O2
3	5.82	1,1-Diisobutoxy-isobutane	1.75	202	C12H26O2
4	6.58	2,5-Methylene-d,l-rhamnitol	0.58	178	C7H14O5
5	7.06	2-Propanone, 1,1-dibutoxy-	0.65	202	C11H22O3
6	7.43	PROPANE, 1,1',1''-[METHYLIDYNETRIS(OXY)]TRIS[2-METHYL-	2.30	232	C13H28O3
7	8.89	3,3-DIMETHYL-4-OXO-HEXANOIC ACID	0.46	158	C8H14O3
8	19.13	Methyl tetradecanoate	0.42	242	C15H30O2
9	23.04	1,2-Benzenedicarboxylic acid,bis(2-methylpropyl) ester	51.02	278	C16H22O4
10	23.11	DIISOBUTYL BENZENE-1,2-DICARBOXYLATE	11.16	278	C16H22O4
11	23.45	Hexadecanoic acid, methyl ester	5.14	270	C17H34O2
12	24.80	n-Hexadecanoic acid	5.60	256	C16H32O2
13	26.33	7-Methyl-Z-tetradecen-1-ol acetate	0.49	268	C17H32O2
14	26.47	9-Octadecenoic acid (Z)-, methyl ester	4.85	296	C19H36O2

15	26.94	Methyl stearate	2.10	298	C19H38O2
16	27.21	Hexadecanoic acid, 2-methylpropylester	1.15	312	C20H40O2
17	27.79	cis-Vaccenic acid	5.25	282	C18H34O2
18	28.19	Octadecanoic acid	2.42	284	C18H36O2
19	30.09	Oleic Acid	0.97	282	C18H34O2
20	30.54	Octadecanoic acid, 2-hydroxy-1,3-propanediyl ester	0.39	624	C39H76O5
21	38.46	tert-Hexadecanethiol	0.44	258	C16H34S

Supplementary Figure S1. The chemical composition of *Asian pigeonwing*. A) HPLC Chromatograph Report. B) GC-TSQ mass spectrometer
